# Pseudoerosions of Hands and Feet in Rheumatoid Arthritis: Anatomic Concepts and Redefinition

**DOI:** 10.3390/jcm8122174

**Published:** 2019-12-09

**Authors:** Lena Hirtler, Claus Rath, Hannes Platzgummer, Daniel Aletaha, Franz Kainberger

**Affiliations:** 1 Division of Anatomy, Center for Anatomy and Cell Biology, Medical University of Vienna, Vienna 1090, Austria; claus.rath@meduniwien.ac.at; 2 Department of Biomedical Imaging and Image-Guided Therapy, Medical University of Vienna, Vienna 1090, Austria; hannes.platzgummer@meduniwien.ac.at (H.P.); franz.kainberger@meduniwien.ac.at (F.K.); 3 Division of Rheumatology, Department of Internal Medicine, Medical University of Vienna, Vienna 1090, Austria; daniel.aletaha@meduniwien.ac.at

**Keywords:** rheumatoid arthritis, pseudoerosions, hand, foot, ultrasonography, radiography, computed tomography, magnetic resonance imaging

## Abstract

Rheumatoid arthritis is a chronic inflammatory disease characterized by the development of osseous and cartilaginous damage. The correct differentiation between a true erosion and other entities—then often called “pseudoerosions”—is essential to avoid misdiagnosing rheumatoid arthritis and to correctly interpret the progress of the disease. The aims of this systematic review were as follows: to create a definition and delineation of the term “pseudoerosion”, to point out morphological pitfalls in the interpretation of images, and to report on difficulties arising from choosing different imaging modalities. A systematic review on bone erosions in rheumatoid arthritis was performed based on the Preferred Reporting Items for Systematic Reviews and Meta-Analyses (PRISMA) guidelines. The following search terms were applied in PubMed and Scopus: “rheumatoid arthritis”, “bone erosion”, “ultrasonography”, “radiography”, “computed tomography” and “magnetic resonance imaging”. Appropriate exclusion criteria were defined. The systematic review registration number is 138826. The search resulted ultimately in a final number of 25 papers. All indications for morphological pitfalls and difficulties utilizing imaging modalities were recorded and summarized. A pseudoerosion is more than just a negative definition of an erosion; it can be anatomic (e.g., a normal osseous concavity) or artefact-related (i.e., an artificial interruption of the calcified zones). It can be classified according to their configuration, shape, content, and can be described specifically with an anatomical term. “Calcified zone” is a term to describe the deep components of the subchondral, subligamentous and subtendinous bone, and may be applied for all non-cancellous borders of a bone, thus representing a third type of the bone matrix beside the cortical and the trabecular bone.

## 1. Introduction

Rheumatoid arthritis (RA) manifests with three types of structural joint damage: joint space narrowing, erosions, and capsular abnormalities in the form of synovial proliferation and subluxations [1,2,3,4]. The diagnosis of erosions and their quantification as part of radiographic scoring systems is an accepted surrogate biomarker of structural progression of arthritis [4,5]. Erosions in RA have been defined in consensus statements and in studies with high-resolution peripheral quantitative computed tomography (HRpqCT) as cortical defects, breaks, or other discontinuities with underlying trabecular bone loss and characteristic locations that can be identified with imaging [6,7,8,9,10,11,12]. On radiographs, according to the 2010 ACR/EULAR (American College of Rheumatology/European League Against Rheumatism) rheumatoid arthritis classification criteria [13,14], erosions have to be seen at least at three separate joints at the interphalangeal (PIP), metacarpophalangeal (MCP), wrist (counted as one joint), or metatarsophalangeal (MTP) joints [15,16]. For ultrasound (US) and magnetic resonance imaging (MRI), the operational OMERACT (outcome measures in rheumatology) definition requests the abnormality being visible in two planes [17,18].

At the wrist, the most frequent locations are the capitate, ulna, lunate, triquetrum, and scaphoid [19,20,21,22,23,24,25,26], at the ankle the distal fibular notch, the navicular, cuneiform and cuboid bones are often involved, the talus and calcaneus less frequently [27,28]. Why erosions occur at these sites is commonly explained by immunological and anatomical models [29,30,31]. The latter mainly refer to the thinning of cartilage near capsular insertions at bones (bare areas) and to microdamage [32,33,34,35,36]. Following immunologically-based concepts, erosion formation is explained by increased bone resorption and decreased bone formation at certain locations in the subchondral bone [37]. Werner et al. [32] showed a correlation between cortical micro-channels and the occurrence of bone erosions in bare areas.

Especially in early, preclinical or undifferentiated arthritis with small or no erosions, it is necessary to differentiate a true rheumatic erosion from the various forms of normal erosion-simulating concavities of the bony surface and therefor avoid false-positive statements [38,39]. Such so-called pseudoerosions [40] have been described to be smooth and well demarcated on radiographs, ultrasound, computed tomography (CT) and MRI [41]. The effect of misinterpreting a normal anatomic concavity as an erosion or vice versa may be estimated from the intra- and inter-reader variations of scoring systems and has been directly mentioned for the RAMRIS (rheumatoid arthritis MRI score) [42,43]. The spectrum of MRI “erosion-like” lesions is broad: Ejbjerg et al. [44] observed them in 1.9% of healthy persons, whereas Olech et al. [45] saw them in 65%. Rothschild [46] questioned if such findings should be interpreted as true erosions, old erosions from earlier diseases without clinical significance, or other. For the US, a 30% false-positive rate of erosion detection has been reported [47]. The computer-assisted assessment of erosions was considered helpful, but difficulties in discriminating those from normal bony concavities were observed [48,49].

The aim of this systematic review was (1) to evaluate the frequency of specifically stated difficulties arising in the interpretation of imaging modalitis in search for bone erosions, (2) to define the characteristic anatomic appearances and patterns of pseudoerosions with respect to the potential pitfalls in the diagnosis of RA as reported in the literature and (3) to develop an anatomic concept for improving the accuracy and precision of imaging assessment.

## 2. Materials and Methods

A systematic review on bone erosions in RA was performed based on the guidelines of the PRISMA (Preferred Reporting Items for Systematic Reviews and Meta-Analyses) statement and was registered accordingly (No. 138826) [50].

### 2.1. Search Strategy

The search was performed in PubMed (Medline) and Scopus with the following search terms: “rheumatoid arthritis”, “bone erosion”, “ultrasonography”, “radiography”, “computed tomography” and “magnetic resonance imaging” (example for search in PubMed: “rheumatoid arthritis” AND bone AND erosion AND (ultrasonography OR radiography OR “computed tomography” OR “magnetic resonance imaging”). No specific date was defined as starting point, the end of search was 31 May 2019. English language was defined as a required criterium.

### 2.2. Selection Criteria

All original studies investigating the diagnosis of RA with X-ray, sonography, CT or MRI and describing false positive diagnoses of bone erosions and erosion-like changes published before 31 May 2019 were included. Exclusion criteria were animal studies, feasibility studies, other inflammatory diseases, clinical studies comparing therapeutic measurements in RA, studies comparing the sensitivity of imaging modalities without report of false positive diagnosed erosions or erosion-like lesions, surgical procedures or longitudinal studies without direct reference to this topic, case reports and conference papers. Additionally, all papers without full text availability were excluded from the analysis.

Data extraction was performed by using a standardized Excel (Microsoft Corporation, Redmont, WA, USA) data extraction form: first author, year of publication, country, study population, number of patients, imaging modality, joints evaluated, reported sensitivity of imaging modalities, reported false positive or false negative diagnosis of bone erosions, reported limitations in image interpretation with respect to anatomy, the differential diagnosis to other erosive diseases, artifacts and signal-to-noise reduction.

## 3. Results

**Figure 1:**
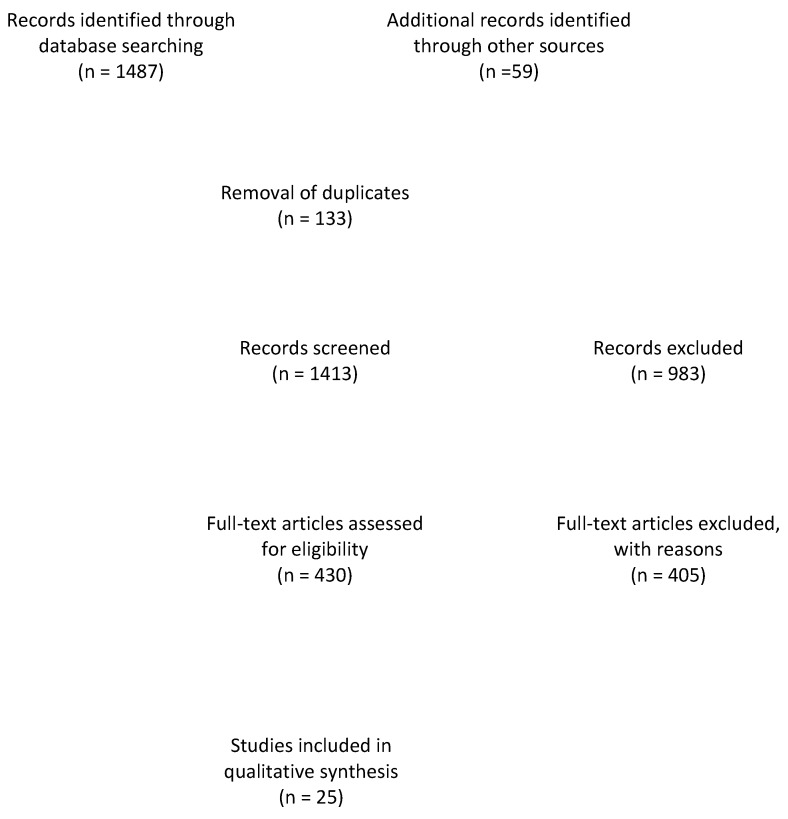
Flow diagram of the literature review.

The search with the defined terms resulted in a total of 1487 results. An additional number of 59 papers were added after reference-screening. The flow diagram of the literature review may be seen in Figure 1. Ultimately, only 25 papers reported specifically on false-positive results or erosion-like changes.

Based on the information gathered in the remaining papers, the false-positive results were subdivided into anatomic pseudoerosions, if the explanation for the false-positive diagnosis was described as a morphological phenomenon, and into artifact-related pseudoerosions, if the explanation for the false-positive diagnosis was related to the respective imaging technique.

### 3.1. Anatomic Pseudoerosions

**Figure 2:**
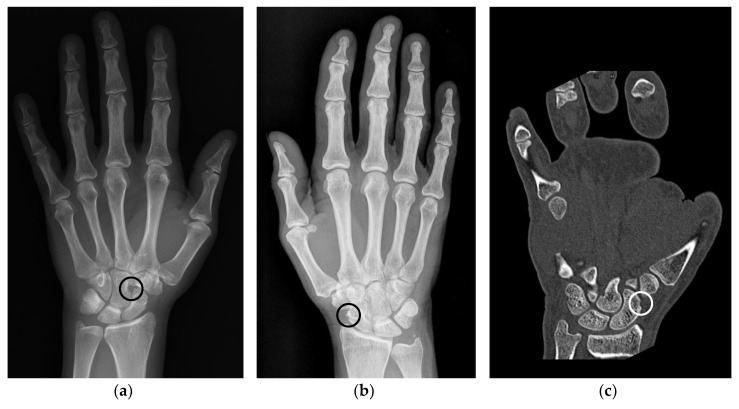
Examples of anatomical pseudoerosions. (**a**) Example of a sulcus like pseudoerosion of the capitate bone (black circle) in a left hand of a 52 years old female patient. Referred for suspected scaphoid fracture, which was not verified. (**b**) Example of a pseudoerosion at the level of the scaphoid waist (black circle) in a right hand of a 66 years old female patient. Referred because of unspecific wrist pain, which afterwards subsided without treatment after one week. (**c**) Scaphoid rim simulating an erosion in a left hand of a 38 years old male patient (white circle). Referred because of presurgical planning after fracture of the fifth metacarpal and luxation of the fourth and third metacarpal.

Anatomic pseudoerosions, i.e., normal concavities of a bone with a potential for misinterpreting them as arthritis-related erosions, were described in twelve original papers and reviews and may be classified into four types according to their anatomic form and configuration (Table 1): (1) a groove or notch or its incomplete form, i.e., a jutty, (2) a sulcus as part of an osteofibrous channel, (3) a subcapital neck on long bones, or (4) a nutritional channel or a zonal roughness [3,11,41,51,52,53,54,55,56,57]. According to their shape, they may be grouped into (1) shallow or broad concavities and (2) subchondral cysts, if en-face displayed on an image and occasionally with a small opening to the joint space, or (3) channel-like structures (Figure 2a,c) [3,54,55]. The anatomic location of pseudoerosions is predominantly at the carpal bones, the MCP- and the MTP-joints. Almost always they are linked to a ligament insertion (Figure 2b), a mucosal fold fixation or the hood of a tendon sheath, and occur at the noncortical bone, also known calcified zones (i.e., borders of the subchondral and enthesial calcified bone with the adjacent underlying trabecular structures). The content of pseudoerosions is visible with US and MRI and may be normal or degenerated ligament tissue, or blood vessels [44,56] and the development of edematous changes [58]. With contrast media, a slight enhancement can be observed, however, in one publication rare cases of strong enhancement was documented [56].

### 3.2. Artifact-Related Pesudoerosions

Artifact-related pseudoerosions were mentioned in 18 original papers and reviews and may be caused due to (1) partial volume artifacts of cross-sectional images or other modality-specific artifacts (ultrasound diffraction or reflection, insufficient fat suppression with MRI), or (2) a low signal-to-noise ratio (Table 2) [1,5,52,57,58,59,60,61,62,63,64,65,66,67,68].

## 4. Discussion

From the viewpoint of imaging anatomy, a misinterpretation of erosions in RA may occur due to (1) anatomic pseudoerosions, or (2) artifact-related pseudoerosions as a result of an inadequate investigation technique. Pseudoerosions and erosions are commonly located at certain areas of the surface outline of the calcified bone, also known as calcified zones. These may therefore, besides cortical bone and trabecular bone, be regarded as a third type of organization of the bone matrix.

**Figure 3:**
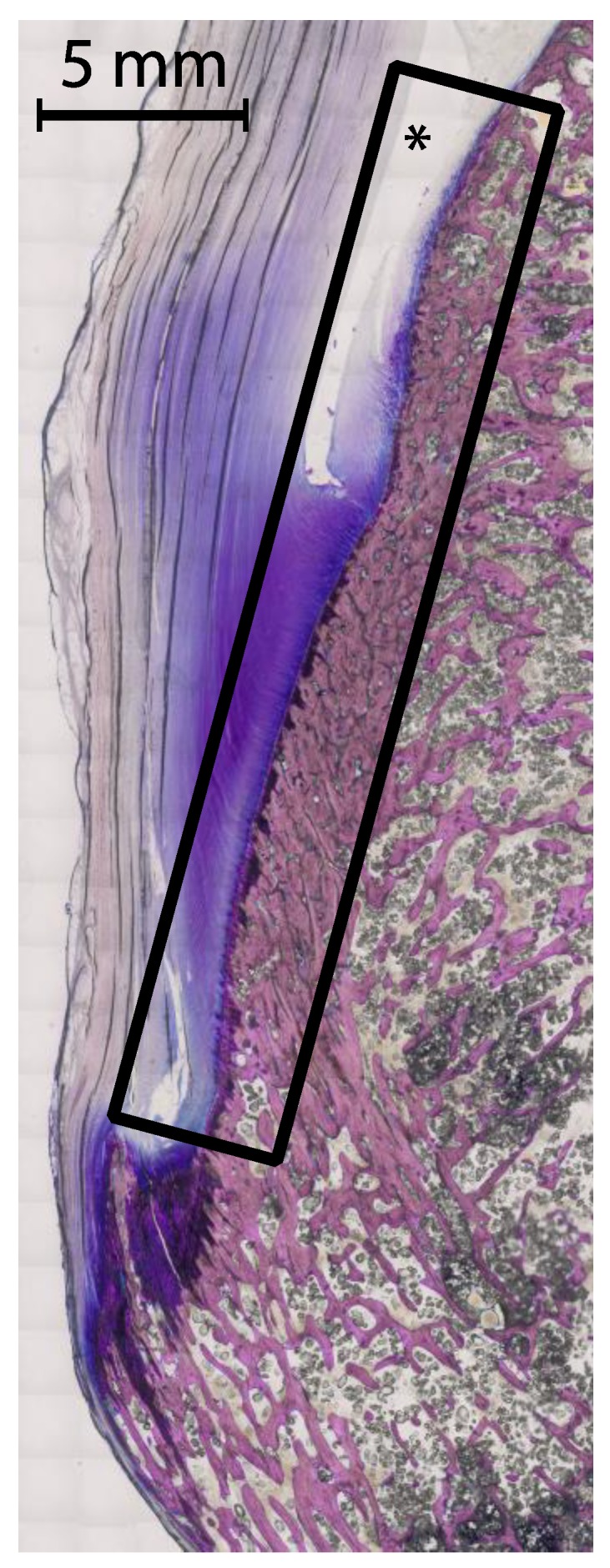
Example of a calcified zone. Thin ground section of the calcaneal tuberosity, the calcaneal tendon and the calcaneal bursa—also a frequent location of bone erosions. The described calcified zone as subchondral and enthesial calcified bone with adjacent underlying trabecular structures including the overlying tissues is marked by the rectangle. The asterix marks the calcaneal bursa. A 5 mm scale is included, the tissue was stained with Giemsa.

The term “calcified zones” (Figure 3) in this context is therefore proposed to describe the borders of the subchondral and enthesial calcified bone with the adjacent underlying trabecular structures. It may be extended for describing all parts of intraarticular bone apart from the cortex. With its overlying tissue of hyaline cartilage, synovium or capsule-ligamentous structures it forms anatomic units. The relationship between these zones and the adjacent tissues is so tight that the fibrous layers of tendon sheaths, bursae, periosteum or the cartilaginous zones of entheses or hyaline cartilage are in direct continuation with the subjacent bone, thus providing direct contact with synovial tissue. The concept of the subchondral zone was used by Dihlmann [71] to describe the mineralized zone of hyaline cartilage as part of the subchondral bone. It may be extended to describe a subligamentous, subtendinous or subbursal zone of the bone. Utilizing sub-millimeter spatial resolution CT, these calcified zones can be displayed. Differentiating the normal calcified zone from erosional changes, i.e., irregular margins and sclerotic reaction, is the main challenge in differentiating true erosions from pseudoerosions [72].

Pseudoerosions have to be differentiated from other pathologies as ganglion cysts, crystal-induced arthropathies, tuberculosis or other infections, and from degenerative lesions in the form of erosions, subchondral (pseudo)cysts or beak-shaped osteophytes as there are so many similarities in location [38,60]. Intraosseous ganglion cysts are common and almost always have a continuity with a ligament which underwent mucous degeneration [73,74]. Especially in the elderly population, the more prevalent degenerative changes of the bone may be difficult to be differentiated from RA-related erosions [38,75]. However, in children interpretational problems may arise. There, normal concavities simulating erosions have been referred to as “bony depressions” at certain locations in the wrist [76,77,78]. Such pseudoerosions in children may be big, indicating that size is not a reliable feature for differentiating normal variants from true erosions.

### 4.1. Anatomic Pseudoerosions

**Figure 4:**
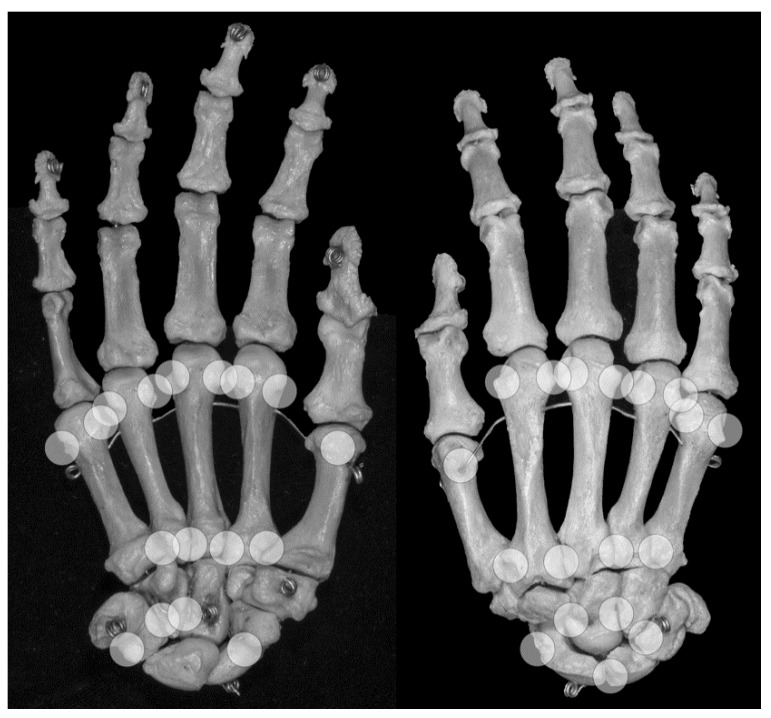
Locations of anatomical pseudoerosions. Overview on possible locations of anatomical pseudoerosions as summarized in Table 3. Right skeletal hand, on the left view from palmar, on the right view from dorsal.

An anatomic pseudoerosion can be defined as a normal concavity of a bone outlined by a smooth and thin calcified zone with the potential for a false-positive misinterpretation of an erosion. In this form, the term pseudoerosion is more precise than “notch” or “bony depression” and may be preferred as it contains a prognostic impact for the imaging assessment of arthritis. Such clinically oriented annotations, examples are the scaphoid waist and the metacarpal neck as typical sites for fractures, have been in use in traumatology and may be of help in the assessment of arthritis-related erosions, too (list of described pseudoerosions in Table 3, an overview of anatomical pseudoerosions in the hand may also be found in Figure 4).

Grooves due to ligament or tendon insertions have a varying appearance as described in the enthesis concept by Benjamin and McGonagle [79]. Such prominent grooves can cause the appearance of a pseudoerosion (Figure 2a). A groove may occur in three forms: (1) at a non-apophyseal direct tendon or ligament attachment where the uncalcified components of the enthesis enters the bone, (2) at an apophysis with overhanging edges, or (3) at an incomplete apophysis, a jutty, at the indirect attachments of a tendon or ligament with a tangential transition into the periosteum. For example, pseudoerosions resulting from the first form are the metacarpal ligament insertions at the bases of the metacarpal bones [80]. At the dorsal aspect of the triquetral bone, such a pseudoerosion may be formed by the distal insertion of the radiotriquetral ligament along with other components of the dorsal radiocarpal ligament. On the capitate, on which several strong carpal ligaments have their insertion, and many other carpal bones, intercarpal ligaments may cause pseudoerosions [51]. Examples for the second form may be the non-spherical form of metacarpal and metatarsal heads, which can be explained by the collateral ligament complexes running laterally and medially with smoothly outlined shallow metacarpal grooves containing these structures. At the metacarpals, these grooves are bordered by little tubercles for the proximal attachment of the collateral ligaments (Figure 4) [81]. Moraes do Carmo et al. [54] identified three concavities in the first metacarpal head (intersesamoid, ulnar, and radial) and two in those of the fingers (ulnar and radial). They described dorsal depressions of the metacarpal heads due to the extensor digitorum tendons in one third of their anatomic specimens which correlated with observations with ultrasound made by Boutry et al. [82,83]. A similar study was done for defining pseudoerosions of the metatarsal heads by Torshizy et al. [55] who described anatomic variations in the normal osseous concavities of the lateral and medial aspects of each metatarsal head. Typical jutties, i.e., examples for the third form of grooves, are the small round or oval subcapsular notches at the proximal phalangeal bases [80,84]. At the Achilles tendon insertion, proximal to its jutty shallow irregularities beneath the calcaneal bursa may represent true erosions [85].

Osseous sulci are commonly roofed with a ligament, fascia or other fibrous tissue, thus forming an osteofibrous channel for a tendon within a synovial tendon sheath. A subcapital neck of the distal metacarpal and the metatarsal bones is a small metaphyseal narrowing that may cause a pseudoerosion on projection radiographs, ultrasound or MRI [86]. At the distal fifth metacarpal bone, due to its slight varus angulation this neck may be more prominent.

Nutritional channels may appear as pseudoerosion on MR if their orifice is displayed as a little T2-weighted hyperintense spot [51]. Their superficial orifice is often located at a roughness of the calcified zones which as a whole may simulate an erosion [11,34,87]. Some of these iuxtaarticular surface roughnesses may be specified as crests or ridges that correspond to attachment sites for redundant joint capsule [55]. Others, especially on carpal bones, may be due to indentations of innominate ligamentous attachments or synovial folds [51]. Such typical structures visible between the radial aspect of the scaphoid and the radial carpal collateral ligament may be called scapho-capsular ligaments (Figure 2C). Roughness of the calcified zones may be visible at various sites and should be differentiated from shallow extensive true erosions and from advanced cartilage degeneration [88].

### 4.2. Artefact-Related Pseudoerosions

Artefact-related pseudoerosions are defined as an interruption of the sharp outline of the calcified zones. Important causes are a low signal-to-noise ratio, a partial volume artefact, or in case of ultrasound irregular backscattering with artefacts on an incongruent or rough surface. A low signal-to-noise ratio could be caused by over-penetration of the X-ray beam through the bone or due to insufficient spatial or contrast resolution. This effect is more severe in cases with low calcium content in the calcified zones or the subjacent trabecular bone, previously referred to as subchondral osteoporosis or as pre-erosions, and may be enhanced by swelling of the overlying soft tissue. With ultrasound, diffraction or a complex backscattering of the waves on a curved or irregular surface may cause various pseudo-effects on the retrieved image [51,69,89].

Although X-ray is most commonly used in the diagnosis of RA it is CT which can be regarded as the best imaging modality for differentiating pseudoerosions from true erosions [53,62,63,90,91,92,93,94]. Several studies [34,95,96,97,98] describe a significant decrease of trabecular volume and number and an increased trabecular heterogeneity in patients with rheumatoid arthritis by using HRpqCT. This trabecular bone loss as the intramedullary component of bone erosions may contribute the largest part and may therefore be a reason for misinterpretation of erosions or pseudoerosions in radiographs as this imaging method is relatively insensitive to trabecular bone loss [60,99].

In addition, MRI and US are reported to be more sensitive than plain radiography [53,62,90,91,92,93,94], but this especially seems to be dependent on the location investigated [88,100]. In some cases, radiography may even be superior to MRI in detecting bony erosions despite its lack of three dimensionality [1,3,5,58,99,101,102]. Through its high spatial resolution it can differentiate smaller erosions which otherwise would present themselves as continuous on MRI [61,65].

Thus, it is important to recognize several parameters to achieve a decrease of cognitive diagnostic errors especially in early arthritis. These include slight variations in the respective projection technique and individual ligament laxity or postinflammatory scarring of ligaments. In addition, the roughness of a calcified zone, and the transitional changes between normal bone and true inflammatory erosions are until now not or only scarcely addressed. Even the projection of the joints, even if the relevant anatomic landmarks are displayed according to the standards, is highly variable. One has also to keep in mind that discrete forms of malalignment due to ulnar deviations or other forms of arthritic subluxation, ligament laxity with a slight rotation of bones, and variations in their arrangement may cause a more prominent appearance of a pseudoerosion [3,51,57,102].

### 4.3. Erosions-in-Pseudoerosions

**Figure 5:**
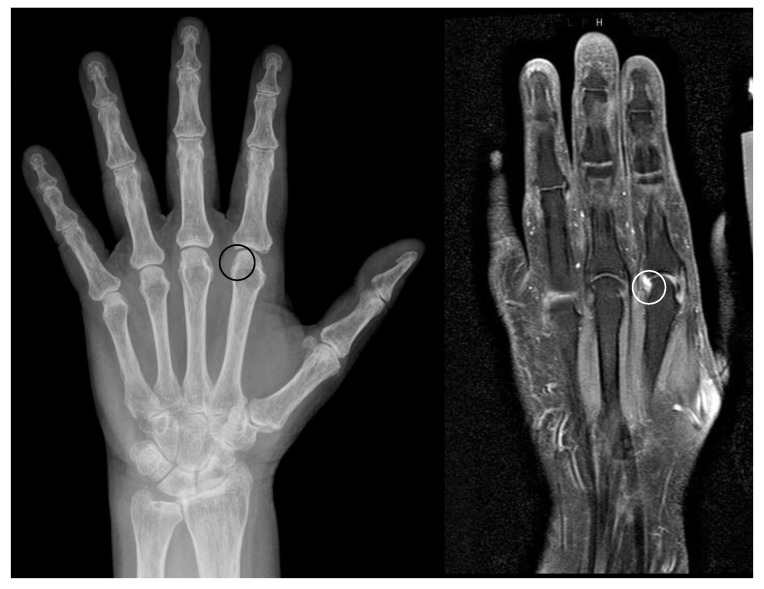
Erosions within pseudoerosions. Example of the development of an erosion within a pseudoerosion (black/white circle) in a left hand of a 65 years old female patient with longstanding mild seropositive rheumatoid arthritis. Left: radiograph, right: MRI.

Both anatomic and artefact-related pseudoerosions are located at sites with direct or indirect contact to inflammatory tissue in arthritis, and therefore, are at higher risk for destruction. Areas of the articular bone without any cartilage covering are more prone to erosive destructions by synovial tissue and effusion [3,32]. Hence, in an anatomically preformed concavity a true inflammatory erosion may develop. McQueen et al. [51] described these erosions-in-pseudoerosions (Figure 5) for the attachment sites of the intercarpal ligaments. It may be observed at the site of ligamentous attachments covered by synovial folds at the metacarpal or metatarsal heads or at the wrist. On the other hand, true erosions may be classified as normal variants. It remains unclear whether these are incidental findings or subclinical erosions [3,57,102].

### 4.4. Limitations

A limitation of this study was that the defined search terms resulted in a large quantity of papers, which had to be screened. However, generally accepted terms for mimickers of true erosions do not exist, are described in various forms and additionally with more equivocal definitions than expected at the beginning of this project. Nonetheless, this wide search net allowed for the inclusion of all relevant sources describing the phenomenon of pseudoerosions and minimized the possibility of excluding the respective publications.

## 5. Conclusions

In conclusion, a pseudoerosion is more than just a negative definition of an erosion. It can be defined as a normal osseous concavity (anatomic pseudoerosion) and/or an artefactual interruption of the calcified zones (artefact-related pseudoerosion). It can be classified according to their configuration, shape, content, and can be directly anatomically named. “Calcified zone” is a term to describe the deep components of the subchondral, subligamentous and subtendinous bone and may be applied for all non-cancellous borders of a bone, thus representing a third type of the bone matrix beside the cortical and the trabecular bone. Anatomic pseudoerosions are almost always related to a ligament insertion or the osteo-fibrous channel of a tendon sheath, therefore, being of high risk for microdamage and the development of a “true” arthritic erosion. Understanding these peculiar aspects of the bony surface with relation to ligament insertions and osteofibrous channels may be of help in improving the assessment of erosions and for reducing over- and underdiagnosis of true erosions.

## 6. Take Home Message

1. Pseudoerosions may be subclassified into anatomic (normal osseous cavity) and artefact-related (artefactual interruption of the calcified zone).
2. The term “calcified zone” describes the deep components of the subchondral, subligamentous and subtendinous bone and may be applied for all non-cancellous borders of a bone.
3. Pseudoerosions can be regarded as anatomic sites at risk for the development of “true” arthritic erosions.

## Figures and Tables

**Table 1:** Pseudoerosions.

Citation	Type of Article	Imaging Modality	Reported Pseudoerosion	Explanation
Alasaarela et al., 1998 [58]	Original research	Magnetic resonance imaging (MRI) (1.0T T1, T2 and proton density, PD)	False positive interpretation	Pre-erosive oedematous changes in subchondral bone in MRI
Barnabe et al., 2016 [11]	Original research	High-resolution peripheral quantitative computed tomography (HRpqCT)	Carpal pseudoerosions	Arterial foramina
Canella Moraes Carmo et al., 2009 [54]	Original research	Computed tomography (CT)	Carpal pseudoerosions	ligament insertions tendinous sulci
Dohn et al., 2006 [53]	Original research	Sonography	Erosion-like changes	Metacarpophalangeal (MCP) joints
Dohn et al., 2013 [57]	Original research	Sonography	False positive interpretation	Cortical irregularities (osteophytes, notches at the metacarpal neck, subcortical bone cysts)
Ejbjerg et al., 2004 [44]	Original research	MRI (1.0 T1 spin echo, STIR, T2 spin echo fat-suppressed	Erosion-like changes	Capitate, lunate
Martel et al., 1965 [3]	Original research	Plain radiography	Carpal pseudoerosion	Normal deep groove in the capitate in about 10%
McQueen et al., 2005 [51]	Review article	MRI (T1, T2 fat-saturated)	False positive interpretation	Attachments of interosseous ligaments of the wrist, articular ligaments of the MCP joints, nutrient foramina
Peluso et al., 2015 [52]	Original research	3D sonography	False positive interpretation	Arterial foramina Osteophytes
Robertson et al., 2006 [56]	Original research	MRI (1.5T, T1 spin echo, fat-suppressed FSE, fat-suppressed PD-weighted FSE, 3D SPGR)	Carpal pseudoerosions	ligament insertions
Torshizy et al., 2008 [55]	Original research	CT	Tarsal pseudoerosions	attachment site of joint capsule ligament insertions tendinous sulci
Wawer et al., 2014 [41]	Original research	Plain radiography	Carpal pseudoerosions	ligament insertions

STIR = Short TI Inversion Recovery, FSE = Fast Spin Echo, SPGR = Spoiled Gradient Recalled Echo.

**Table 2:** Imaging difficulties.

Citation	Type of Article	Imaging Modality	Reported Problem
Alasaarela et al., 1998 [58]	Original research	CT	Examination of a curvilinear object—the more the reformat plane parallels the z-axis, the more resolution of multiplanar reformats is impaired. The partial volume effect is harmful.
		Plain radiography	Information dependent on projections used
Albrecht et al., 2013 [1]	Original research	Plain radiography	2D character of radiography
		CT	No simultaneous assessment of inflammatory changes of RA
Amin et al., 2012 [62]	Original research	Plain radiography	Beam has to hit erosion tangentially to show cortical break
Aurell et al., 2018 [63]	Original research	Plain radiography	Possibility of false negative evaluation, if the orifice of the erosion is not hit tangentially
Cimmino et al., 2002 [60]	Original research	MRI (T2 spin echo or gradient echo)	Failed fat suppression can mimic bone marrow edema
Dohn et al., 2013 [57]	Original research	Sonography	Some areas of hand and wrist are inaccessible for ultrasound beam
Dohn et al., 2008 [65]	Original research	MRI (0.6T T1 3D fast field echo)	Overestimation of erosion size due to difficult differentiation between cortical bone and erosion
Ejbjerg et al., 2006 [64]	Original research	Plain radiography	Up to 30% of an MCP joint bone has to be eroded before detection
Emond et al., 2012 [68]	Original research	MRI (1T 3D spoiled gradient echo)	Boundaries of erosions difficult to differentiate
Foley-Nolan et al., 1991 [59]	Original research	Plain radiography	Erosions only visible when large percentage of bone thickness has been destroyed
Forslind et al., 2003 [61]	Original research	Plain radiography	Delineation of erosions difficult in patients with osteoporosis
		MRI (1.0T 3D T2 gradient echo, T1 spin echo with and without fat-saturation)	False negative interpretation due to contiguous looking erosions
Kleyer et al., 2016 [66]	Original research	MRI (1.5T T1)	Small cortical breaks not seen on MRI—validation by HRpqCT
McQueen et al., 1998 [69]	Original research	MRI (1.5T T1 and T2 with and without fat suppression)	Partial volume artefacts may lead to false positive indications of erosions
McQueen et al., 2001 [70]	Original research	Plain radiography	Identification of erosions hampered by poor visibility at the carpus
Peluso et al., 2015 [52]	Original research	Ultrasonography	Due to anatomical structure, multiplanar distribution of bones that restricts the ultrasound beam and alters the correct visualization
Ulas et al., 2019 [67]	Original research	MRI (1.5T): Susceptibility-weighted imaging, SWI T1w	False positive identification of erosions due to motion artefacts, strong susceptibility artefacts at tissue intersections Weak differentiation of cortical bone
Wakefield et al., 2000 [5]	Original research	Plain radiography	Typical anatomical location of bone erosions difficult to see until it lies in the tangential plane of the radiographic beam.
		Plain radiography	Periarticular osteoporosis
Wawer et al., 2014 [41]	Original research	Plain radiography	Less density in subcortical cancellous bone due to synovial and bony hyperemia, overlapping of carpal bones, presence of osteophytes

**Table 3:** List of pseudoerosions with anatomic description.

Location	Name	Description
Scaphoid waist, palmar aspect	Scaphoid waist	Tendon hood of radial-sided carpal tunnel with radio-scapho-capitate ligament
Scaphoid, radial aspect of midpart		Scapho-capsular ligament or mucosal fold insertions
Capitate, distal ulnar portion [41]	Ulnar capitate notch	Intercarpal ligaments
Capitate, radial portion	Radial capitate notch	Intercarpal ligaments
Lunate, radial aspect		Scapholuntate ligament
Hamate, distal radial portion [41]		Insertion of capitatohamate ligament and carpometacarpal ligaments
Hamate, distal ulnar portion [41]		Insertion of carpometacarpal ligaments
Triquetrum, radial and dorsal aspect	Radial triquetral notch	Insertion of the radiotriquetral ligament
Triquetrum, ulnar and proximal aspect		Insertion of the ulnotriquetral ligament
Metacarpal bases	metacarpal base notches	Insertion of intercarpal ligaments
Metacarpal or metatarsal neck and heads	metacarpal or metatarsal head notch	Insertion of metacarpophalangeal ligaments or joint capsule
5th metatarsal head		Slight normal varus angulation of metatarsal head
Achilles tendon insertion		Insertion jutty
